# Dynamic *Alu* Methylation during Normal Development, Aging, and Tumorigenesis

**DOI:** 10.1155/2014/784706

**Published:** 2014-08-27

**Authors:** Yanting Luo, Xuemei Lu, Hehuang Xie

**Affiliations:** ^1^Beijing Institute of Genomics, Chinese Academy of Sciences, Beijing 100101, China; ^2^Epigenomics and Computational Biology Lab, Virginia Bioinformatics Institute, Virginia Tech, Blacksburg, VA 24060, USA; ^3^Department of Biological Sciences, Virginia Tech, Blacksburg, VA 24060, USA

## Abstract

DNA methylation primarily occurs on CpG dinucleotides and plays an important role in transcriptional regulations during tissue development and cell differentiation. Over 25% of CpG dinucleotides in the human genome reside within *Alu* elements, the most abundant human repeats. The methylation of *Alu* elements is an important mechanism to suppress *Alu* transcription and subsequent retrotransposition. Decades of studies revealed that *Alu* methylation is highly dynamic during early development and aging. Recently, many environmental factors were shown to have a great impact on *Alu* methylation. In addition, aberrant *Alu* methylation has been documented to be an early event in many tumors and *Alu* methylation levels have been associated with tumor aggressiveness. The assessment of the *Alu* methylation has become an important approach for early diagnosis and/or prognosis of cancer. This review focuses on the dynamic *Alu* methylation during development, aging, and tumor genesis. The cause and consequence of *Alu* methylation changes will be discussed.

## 1. Introduction

According to the most recently annotated human genome (hg19), there are approximately 5.3 million copies of repetitive elements, tandem or interspersed, constituting 50.6% of the human genome [1]. The most abundant repeats are the* Alu* repeats of short interspersed repetitive elements (SINEs) which comprise 13.7% of the human genome. A full-length* Alu* element is approximately 300 nucleotides in length and contains two almost identical monomers separated by an A-rich region. Most of the* Alu* repeats share extensive homology over the 300 nt sequence with a recognition site for the restriction enzyme AluI [2]. The ancestor of* Alu* monomer is the* 7SL RNA* gene, which codes for the RNA component of the signal recognition particle (SRP) involved in the translocation of newly synthesized proteins. Similar to the* 7SL *gene,* Alu* elements with intact internal promoters may be transcribed by RNA polymerase III [3, 4], which is the symbol of the activity of* Alu *elements. Transcribed* Alus* vastly lack important sequence features for retrotransposition [5]. On account of the aid of LINE-encoded retrotransposition machinery,* Alu* transcripts gain mobility and expand in genomes through processes involving reverse transcription and integration [6].

*Alu* retrotransposition is an important molecular evolutionary force shaping the primate genomes [7]. The emergence and evolution of primates coincided with the propagation of* Alu* elements in primate genomes 65 million years ago [8]. A direct consequence of* Alu* retrotransposition is the deposit of approximately 1.2 million* Alu* elements, 10.7% in mass, to the human genome. Based on the evolutionary history, these* Alu* elements can be classified in three major subfamilies,* AluJ*,* AluS,* and* AluY* [9]. Among these* Alu* subfamilies, the youngest* Alu *elements,* AluY* and its variants* AluYa-k*, remain very active and exhibit the highest rate of retrotransposition in the human genome [10, 11]. A study of comparative genomics between the orangutan and primate genome revealed that a pristine shared insertion with three orangutan-specific and three human-specific mutations in two lineages may represent an ancient backseat driver of* Alu *element expansion [12]. Recently, distinct events of ectopic* Alu*-*Alu *recombination were detected in the origin of* AluS* and* AluY* source genes [13]. Despite the fact that several recent studies demonstrated that LINE-1 elements contribute substantially to the structural variations in the human genome [14–16], the retrotransposition rate of* Alu *elements is estimated to be one in 21 births, which is ten times higher than that of LINE-1 [17].

**Figure 1:**
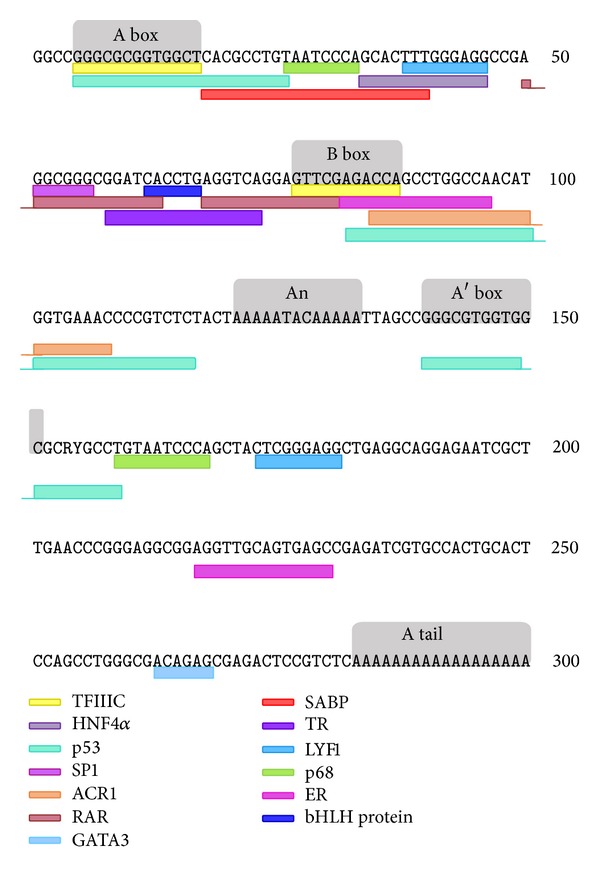
The binding sites of regulatory elements along the* Alu* consensus sequence. The consensus* Alu* sequence is taken from Hambor et al. [133]. The binding motifs are color coded and shown in frames.

Three-decade studies revealed* Alu* elements have a profound impact on the human transcriptome.* Alu* elements harbor sequence motifs for a number of transcription factors (Figure 1), which regulate the expression of* Alu* themselves or their adjacent genes. The presence of* Alu *elements in the human transcriptome can be classified into two forms: embedded* Alu *RNA and free* Alu *RNA [18]. Since a large number of* Alu *elements are present inside genes,* Alu *elements may be transcribed by RNA polymerase II as embedded* Alu *RNA in mRNA transcripts. The embedded* Alu *elements may contribute to alternative splicing sites [19, 20] or polyadenylation signals [21] and thus promote the transcriptome diversity, for example, the formation of circular RNAs [22]. Pairs of inverted* Alu *repeats embedded within one RNA transcript may form double strand structures and regulate gene expression [23, 24]. It has been estimated that approximately 90% of adenosine-to-inosine (A-to-I) RNA editing, a posttranscriptional processing event, occurs in such* Alu *dsRNA [25–27]. Additionally, RNA editing in non-*Alu *regions was found to be dependent on nearby edited* Alu *sites [28]. These* Alu*-mediated events were found to cooccur within the same transcripts modulating transcriptional response, such as apoptosis and lysosomal processes [29]. The transcription of free* Alu *RNA is initiated from an internal Pol III promoter in the left arm of* Alu *element. Recently, the expression of an* Alu*-like noncoding RNA (NDM29) was found to be driven by upstream type 3 Pol III promoters. The NDM29 transcripts contain an entire* AluJb* sequence with a defective internal promoter. Interestingly, this* Alu*-like noncoding RNA promotes cell differentiation and reduces the malignancy of neuroblastomas [30]. Some ncRNA transregulate their target genes depending on* Alu *motifs, for example, ANRIL effects on Chr9p21 [31]. The cellular functions of free* Alu *RNA have been extensively reviewed elsewhere [18, 32–34]. In this review, we concentrate on the dynamic* Alu *methylation during tissue development in aging and tumors.

## 2. Approaches for Global and Genome-Wide* Alu *Methylation Profiling

**Figure 2:**
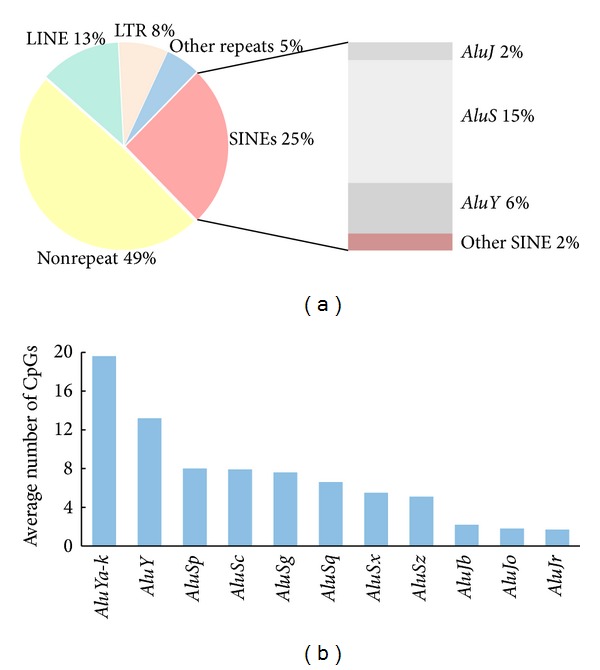
The genome-wide distribution of CpG dinucleotides. (a) Repeats contribute over 50% of CpG dinucleotides in the human genome. The distributions of CpG dinucleotides were calculated based on the hg19 reference genome sequences downloaded from the UCSC database. (b) The average CpG number in an* Alu* element varies between* Alu *subfamilies. The *x*-axis lists the* Alu* subfamilies, among which* AluYa-k* includes* AluYa*,* AluYb*,* AluYc*,* AluYd*,* AluYf*,* AluYg*, and* AluYk* subfamilies. The *y*-axis shows the average number of CpG dinucleotides in a full-length* Alu* element. Only* Alu* elements with a sequence length equal to or greater than 250 bp were included in analysis.

The term “epigenome” refers to the totality of epigenetic alterations occurring on a genome-wide scale [35]. Epigenetics are the additional layers of heritable information occurring at a particular locus that are not directly encoded by the DNA sequence itself but rather involve chemical alterations of chromatin in the form of DNA methylation and histone modifications. The major target for DNA methylation in the human genome is cytosine within the context of CpG dinucleotides. Based on the reference sequences (hg19), there are 28,299,634 CpG dinucleotides in the human genome. Repeat elements encompass more than half of these CpG dinucleotides. The three major repeat families, SINE, LINE, and LTR, contribute 25%, 13%, and 8% of CpG dinucleotides, respectively. Human* Alu *elements (the principal SINE) contribute over 7.1 million CpG dinucleotides, which corresponds to over 23% of all CpGs in the genome (Figure 2(a)). Considering the variation of copy numbers, the average numbers of CG dinucleotides are also shown for* Alu *subfamilies (Figure 2(b)). Apparently, a “younger”* Alu *repeat has many more CpG dinucleotides than the “older” ones.

Since* Alu *elements host one-quarter of CpG dinucleotides in the genome, several techniques have been exploited to ascertain the methylation status of* Alu *repeats as a representation of global methylation level, the average methylation level of the entire genome. Prior to the emergence of PCR,* Alu *specific probes were frequently used in southern hybridization with the genomic DNA predigested with methylation-sensitive restriction endonucleases to determine the methylation statuses of* Alu *subsets [36]. During the last decade, several bisulfite PCR techniques with degenerated* Alu *primers, including* Alu *pyrosequencing [37] and the methylight assay [38], have been applied to provide a quantitative means to assess the methylation levels of targeted CpG dinucleotides in* Alu *subsets. For the intra-*Alu *PCR, both PCR primers were designed with an* Alu *consensus sequence to target a CpG rich segment. Due to the high sequence similarity among* Alu *elements, thousands of* Alu *elements could be amplified simultaneously with one primer set. However, the high frequency of deaminated CpG dinucleotides in* Alu *elements often causes significant signal compression. Since more than 70% of* Alu *elements are with CA or TG instead of CpG shown in* Alu *consensus sequence, using primers designed with* Alu *consensus sequence, the detected methylation level for a completely methylated genome would be less than 30% [37]. The sensitivity of* Alu *pyrosequencing or* Alu *methylight assay could be improved by using primers designed against CpG rich* Alu *elements [38].

Recently, two genome-wide strategies were developed to target the methylation statuses of a great number of* Alu *elements. Since most* Alu *elements are heavily methylated in somatic tissues, the identification of unmethylated* Alu *elements will provide clues for the* cis-* or* trans-*factors associated with* Alu *methylation. To track the unmethylated* Alu*, Rodriguez and colleagues exploited a methylation-sensitive restriction enzyme SmaI (recognition size CCCGGG) to digest genome DNA and release the* Alu *elements containing an unmethylated SmaI site [39]. Based on this approach, it was estimated that approximately 2.3% of* Alu *elements would be hypomethylated in normal tissues. In addition, the highest methylation rate was observed for the youngest* AluY* subfamily [39]. To generate a genome-wide* Alu *methylation map at single base resolution, an* Alu *anchored bisulfite PCR sequencing approach (AABPS) was developed [40]. In this approach, the genomic DNA was first digested with the methylation-insensitive enzyme AluI, ligated to an adaptor, and then subjected to bisulfite conversion. With a primer specifically designed to target CpG rich* AluY* elements, thousands of* Alu *elements could be amplified simultaneously. According to the* Alu *methylation map generated for normal cerebellum, over 75% of young* Alu *elements were found to be with a completely methylated 5′-end and less than 2% were completely unmethylated. Interestingly, some* Alu *elements exhibited a striking tissue-specific methylation pattern. In addition, the methylation level of* Alu *elements is high in the intronic and intergenic regions, but low in the regions close to transcription start sites [40]. In summary, the age of the* Alu *element and the* Alu *location including nearby genomic features were important factors in determining the extent of* Alu *methylation [39, 40].

## 3. Dynamic* Alu* Methylation during Development and Aging

During mammalian development, extensive genome-wide epigenetic reprogramming occurs at two distinct phases: first during gametogenesis and subsequently at preimplantation [41]. The most complete information about mammalian methylation reprogramming is derived from the mouse model. The early germ cells, or so-called primordial germ cells (PGCs), arise in the posterior primitive streak between mouse embryonic days E6.5 and E7.5 [42]. From E9.5 to E11.5, PGCs leave the gut and migrate into the genital ridge [43]. During the migration into the genital ridge, the genomes of primordial germ cells lose methylation until reaching the gonad. A recent study using bisulfite high-throughput sequencing revealed that the average methylation levels of female and male PGCs at E13.5 are only 7.8% and 16.3%, respectively [44]. Except for intracisternal A particles (IAPs), an active retrotransposon family, all genomic elements including SINE, LINE, and inter-/intragenic regions demonstrate a striking loss of DNA methylation. Interestingly, the methylation level of SINE in the female mouse PGCs is lower than that in male PGCs. PGCs gain parental imprinted regions through* de novo* methylation at male gonadal sex determination or female primordial follicle formation [45, 46]. In early embryogenesis, the paternal genome is actively demethylated soon after fertilization and prior to DNA replication, while the maternal genome is passively demethylated with cleavage divisions [47]. This demethylation process continues up to the 16-cell stage [48]. Prior to implantation,* de novo* DNA methylation occurs in inner cell mass cells to establish the specific methylation patterns of principal cell lineages in early embryos. Such extensive methylation reprogramming processes were also observed in other mammals, including human beings, but the timing and scope of methylation changes may vary [48–51].

Although* Alu *methylation statuses during the two waves of epigenetic reprogramming are still largely unknown, some early studies demonstrated the development of methylation changes of* Alu *elements could be remarkable [52–54].* Alu *elements are heavily methylated in somatic tissues, but decreased approximately 30% in sperm DNA [53]. Decreased methylation levels of* Alu *repeats were also observed in seminoma derived from primary spermatocytes and normal testis which comprises germ cells in all developmental stages [54]. A similar observation was made in the rhesus monkey [52]. The methylation levels of* Alu *elements in primate sperm and seminomas are significantly lower than those in the oocytes and ovarian dysgerminomas, which are primary germ cell tumors (female counterpart of seminomas in males). The methylation levels of mouse PGCs at E13.5 were found to be as low as 8–16% [44]. Such extensive erasure of DNA methylation may also occur in human PGCs. Considering the human* Alu *elements are undermethylated in both testis and matured sperm, it seems at least subsets of* Alu *elements may be hypomethylated for a long period of time from embryonic PGCs to matured sperm. A sperm specific protein was identified to have the capability of binding to an* Alu *sequence specifically and interfering with* Alu* methylation [55]. Since* Alu *methylation levels were low in normal testis, which contains predominately immature sperm, this sperm specific protein might exist in immature sperm prior to meiosis to prevent the* de novo* methylation which occurs to* Alu *elements in male PGCs. Interestingly, compared to ES cells, fetus, placenta, and PGCs, mouse SINE in sperm demonstrated the highest methylation level [44]. Thus, the remarkable low methylation levels of SINE and the presence of* Alu *specific binding protein during spermatogenesis could be limited to primates.

Recently, Lai et al. have explored the methylomes of B cells at different stages of the humoral immune response [56]. Activated by antigenic stimuli, the naïve B cells proliferate in the germinal-center (GC) and differentiate into plasma cells (PC) generating antibodies to eliminate antigens and memory B cells with fast reaction to a second attack by the same antigen. Drastic methylation changes occur during the proliferation of naïve B cells in the germinal-center, while the methylomes of GC, PC, and memory B cells were highly similar to each other. Dynamic* Alu *methylation, in particular for the ones adjacent to genes enriched in function of immune activation and immune response, contributes significantly to the methylome differences between naïve B cells and GC B cells. Additionally, the loss-of-methylation events preferentially occur at the 5′ and 3′ ends of* Alu *elements and reflect nucleosomes repositioning at* Alu *elements. Considering the widespread nature of* Alu *demethylation in naïve to GC B cell transition upon immune activation,* Alu *elements may actively participate in a global restructuring of chromosomal architecture.

For many tissues, the percentage of 5-methylcytosine in genome DNA decreases over time and such DNA demethylation was shown to be age-related [57–61]. Recently, by examining the repeat methylation in blood DNA from a large cohort of elderly subjects, a strong negative correlation was observed between* Alu *methylation and the age of patients [62, 63]. Decreased* Alu *methylation levels were found in patients with Alzheimer's disease [64], osteoporosis [65], and nonneoplastic lung disease [63]. In addition, the progressive loss of* Alu *methylation was linear and correlated with the time since the first visit. However, weak or no correlation was observed between the age and LINE methylation [62, 66]. More interestingly, loss of* Alu *methylation is most significant at certain ages, ranging from 34 to 68 [66]. Although the functional aspects of age-related genome demethylation are still largely unclear, such decrease in the genome-wide level of DNA methylation has been associated with genome instability [57, 67, 68]. It is noteworthy that recent studies on mouse and rat models demonstrated that the methylation alteration with age is highly tissue dependent [69, 70]. In addition, the genomic features and sequence related attributes have profound influence on local epigenome stability. Intergenic and noncoding elements are more vulnerable to the dysregulation of DNA methylation. In particular, the methylation aberrations were frequently found near genes involved in metabolism and metabolic regulation [69]. However, it remains unknown whether* Alu *elements would demonstrate similar patterns of methylation alterations observed on promoter regions and in animal models.

## 4. Aberrant* Alu* Methylation in Tumors

During tumor development and progression, two distinct but concurrent epigenetic abnormalities are commonly observed: localized hypermethylation and global hypomethylation. Global loss of DNA methylation predominately occurs in the repeat sequences, such as LINE, SINE, and satellite DNA [64, 71, 72]. Such methylation loss in repetitive elements has been found in the benign or healthy tissue adjacent to various kinds of tumors, including breast, gastric, colon, and colorectal cancer [71–75]. Although global hypomethylation is not necessarily present in every tumor tissue, it has been experimentally demonstrated that DNA methylation loss leads to an increased carcinogenic potential. Carrying a hypomorphic* Dnmt1* allele, the mice with the hypomethylated genome would develop aggressive T cell lymphomas with a high frequency of chromosomal aberration [76]. Recently, a tumor suppressor gene,* adenomatous polyposis coli (APC)*, was found to be a regulator of DNA demethylation machinery, and the loss of* APC *gene would lead to global hypomethylation and subsequent tumorigenesis [77]. Carrying mutated* APC *genes, both humans and mice are predisposed to intestinal and mammary tumor development [78–80]. Interestingly, in mouse models derived from the crossing of* Apc*Min/+ mice with* Dnmt1*c/+ mice, hypomethylation was shown to promote colon and liver tumor development in early stages but strongly suppress intestinal tumorigenesis in later stages [81].

**Figure 3:**
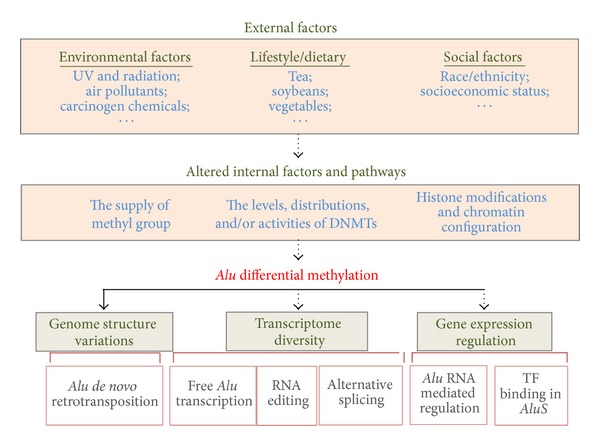
Overview of the potential causes and consequences of differential* Alu* methylation. Differential* Alu *DNA methylation might be induced by various kinds of external factors and mediated with internal cellular factors and pathways. The consequences of differential* Alu* methylation could be multifaceted and shown at three levels: (1) genome structure variations resulted from* de novo Alu *retrotransposition; (2) transcriptome diversity contributed directly by the expression of free* Alu *RNA or indirectly through alternative splicing and RNA editing by embedded* Alu *RNA; (3) gene expression regulations: transcriptional regulation of miRNA/mRNA expression mediated by* Alu *antisense transcripts, duplex structures, or the differential bindings of transcription factors to* Alu *elements. Solid lines represent experimentally validated relationships, while dashed lines are speculative relationships.

Since global hypomethylation is an early event for many tumors, the detection of repeat methylation levels becomes an attractive approach for early diagnosis of cancer. In addition, aiming at cancer prevention, many studies have been conducted to understand the impact of dietary, lifestyle, and environmental factors on global DNA methylation levels (Figure 3). Lam and colleagues revealed DNA methylation is under the influence of demographic, psychosocial, and other factors, such as gender, ethnicity, early SES, total cortisol, and perceived stress [82]. Global DNA methylation level not only depends on the activity of DNA methyltransferases but also relies on the supply of methyl group donors. With DNA methyltransferases, methyl groups could be transferred from S-adenosylmethionine (SAM) to cytosine. Folic acid also known as vitamin B9 is required for the synthesis of SAM. The lack of folic acid in the diet would result in decreased levels of SAM and later global DNA hypomethylation [83]. The inverse association between* Alu *methylation and the gastric cancer risk might be stronger among those who intake more tea, vegetable, folic acid, and particularly isoflavone [84]. Recently, folate supplementation was shown to be sufficient to enhance the DNA remethylation on hypomethylated* Alu *elements and suppress the proliferation of glioma cells [85]. In addition, loss of methylation on* Alu *and/or other repeats has been observed in tissues exposed to radiation [86], UV [87], air pollutants [88, 89], and carcinogen chemicals, including persistent organic pollutants [90, 91]. Interestingly,* Alu *subfamilies show different sensitivity to airborne pollutants and PM10 has a stronger effect on younger* Alus* [92]. A recent study revealed that girls aged 6–17 years with a family history of breast cancer have lower levels of DNA methylation,* Alu *in particular [93]. The race/ethnicity has an effect on* Alu *methylation as well and higher* Alu *methylation was observed among socially advantaged versus disadvantaged groups [94].

Although no direct connection between* Alu* methylation status and carcinogenesis has been established, many studies demonstrated the strong correlations between the global* Alu* methylation level and the clinical outcomes of patients with cancer [95, 96]. A study of mucoepidermoid carcinoma showed an analogously stepwise decrease of* Alu *methylation from the adjacent normal salivary gland to the intermediate, mucous, and squamous cells [97]. Our recent genome-wide methylation study revealed that, during progression of pediatric intracranial ependymomas, the methylation changes are not randomly distributed among* Alu *elements [98]. Compared to normal tissues, only a small set of* Alu *elements gain or lose methylation in tumors. The* Alu *elements proximal to CpG islands tend to be hypermethylated in ependymomas, whereas those in intergenic regions are more likely to be hypomethylated. In addition, the methylation levels of some specific* Alu *elements, either hypermethylated or hypomethylated in tumors, were confirmed to be associated with tumor aggressiveness. Since some* Alu *elements become hypermethylated while some others become hypomethylated, the methylation analysis on a panel of specific* Alu *loci, rather than global* Alu *methylation, could greatly improve the specificity and sensitivity for cancer detection and prognosis. For instance, a study of the* MLH1 *gene in gastrointestinal cancer found that the methylation of an* Alu *element in the first intron spread to its promoter. In normal cells, the spread of* Alu *methylation is limited and a clear boundary is present between that* Alu *element and upstream promoter [99]. The aberrant DNA methylation changes on the promoters or CpG islands have been shown to be tissue specific. However, global methylation changes on repetitive elements are highly prevalent across many cancer types [100]. Whether there is a set of* Alu *elements remains to be addressed, in which methylation levels could be used as common diagnostic or prognostic indicators for a variety of tumors.

## 5. Consequences of Altered* Alu* Methylation Profiles

Since* Alu* elements play important roles in the genome and transcriptome, the consequences of aberrant* Alu *methylation in tumors could be multifaceted (Figure 3). In normal somatic tissues, the CpG dinucleotides within the* Alu* sequence are heavily methylated to suppress* Alu *expression [39, 40, 98, 101], while, in germ cells, sequences adjacent to* Alu *repeats appear to be hypomethylated [102]. In particular, methylation of the* Alu* B box is thought to inhibit RNA Pol III binding and hence block the initiation of* Alu *transcription [103]. Demethylation and consequently transcription of* Alu *elements result in the accumulation of free* Alu *RNA transcripts which may serve as templates for* de novo* retrotransposition [101].* De novo Alu *retrotransposition could be detected by inter-*Alu *PCR which produces multiple bands in a single PCR experiment. Compared to matched normal controls from the same patient, significant alterations in inter-*Alu *PCR profiles were found in nonpolyposis colon tumor DNA samples [104]. In U87MG glioblastoma cells, a significant loss in* Alu *methylation and a subsequent increase in* Alu *transcription were observed under hypoxia conditions [105]. Further comparison of the inter-*Alu *PCR profiles demonstrated an increase in* Alu-*mediated genomic alterations in cells cultured under hypoxia conditions.* De novo Alu *insertion tends to be enriched in high GC content [106]. Interestingly, the loss of* Alu* elements during primate evolution also preferentially occurs in the areas of high GC content [107].

*Alu* repeats possess binding sites of the regulatory elements for their RNA polymerase III transcription (Figure 1). The intact* Alu *elements contain A box and B box bound by RNA polymerase III transcription factor C (TFIIIC) to initiate Pol III-dependent transcription. The binding activity of TFIIIC is the key of the transcription activity of* Alu*. Jang and Latchman found that ICP27 is responsible for the increase in TFIIIC binding and in turn produced the increase in* Alu *transcription [108].* Alu *corepressor 1 (ACR1) [109] and nuclear protein [110] were demonstrated to inhibit Pol III-dependent* Alu *transcription* in vitro*. Sperm* Alu *binding protein (SABP) binding is sufficiently selective to protect* Alu *from* in vitro* methylation without significantly altering the methylation of adjacent CpGs and protect a region (positions 25–33) downstream from the A box promoter element for Pol III [55]. Lukyanov found a narrow binding site of a 68 kDa (p68) protein, a homolog of SABP in the somatic human cell nucleoplasm, and a part of* Alu*-RNA containing nuclear RNP particles [111]. The p68 protein interacts with* Alu *and might participate in* Alu *expression regulation.

Being more than a dramatic source of insertional mutagens, the expression of free* Alu *RNAs may affect nearby gene expression, distal gene expression, and global translation. For instance, the expression of an* Alu *in the promoter of the epsilon-globin gene was found to negatively regulate globin gene expression by transcriptional interference [112]. Recently,* Alu *RNA was found to be a modular transacting repressor of mRNA transcription [113]. Importantly, such transcriptional suppression was found to be specific and limited to certain genes.* Alu *RNAs also affect translational initiation and are found to form stable, discrete complexes with double-stranded RNA-activated kinase PKR and antagonized PKR activation [114]. DICER-dependent fashion mediated free* Alu *RNA processing causes the degradation of a subset of critical stem cell mRNAs (e.g., NANOG) and modulates the exit from the proliferative stem cell state [115]. Meanwhile, DICER1 depletion-induced* Alu *RNA accumulation induces retinal pigmented epithelium cytotoxicity, thus leading to geographic atrophy and blindness [116, 117].

Recently,* Alu *Pol III transcripts have also been associated with the miRNA transcription. Recognized as an important component of the transcriptome, miRNAs are approximately 22 nt noncoding RNAs mediating posttranscriptional regulation [118].* Alu *transcripts are often comprised of 3′-flanking sequence prior to the Pol III transcription terminator “TTTT.” If located within the* Alu *or immediate 3′-flanking regions, miRNAs may be expressed with* Alu *Pol III promoters. Chromosome 19 miRNA clusters (C19MC) were first shown to be transcribed by Pol III with upstream* Alu *promoters. Based on computational prediction and high-throughput small RNA sequencing, an additional twenty-three miRNAs at the downstream of the* Alu *elements were suggested to be transcribed by Pol III with* Alu *promoters [119]. However, the first report for Pol III derived miRNA cluster C19MC has been challenged [120–122]. Recent studies with chromatin immunoprecipitation sequencing of Pol III complexes suggested that some miRNAs, MIR886 [121, 123] and MIR1975 [123], are transcribed with Pol III promoters. According to the UCSC database, no annotated repetitive elements can be found within regions 500 bp flanking the MIR886. An* AluSx* is located in between the spliced MIR1975 but in the antisense orientation with respect to MIR1975 transcription. Thus, the following remains an open question: how frequently and under what condition would miRNA be transcribed with* Alu *promoters?

In addition to being part of the transcriptome, many *Alu* elements may serve as *cis*-regulatory elements in the control of neighboring gene expression. Based on the analysis of the *Alu* consensus sequence, *Alu* elements contain transcriptional factor binding sites for the zinc-finger Pol II transcription factors SP1 and YY1, activator and repressor, respectively, in a sequence-specific binding manner [124] (Figure 1). Based on ChIP-PET data generated with a combination of chromatin immunoprecipitation and paired-end tags sequencing methods, approximately 15% of p53-binding sites were found to reside within *Alu* elements [125]. Apart from those sites preexisting in the progenitors of several *Alu* subfamilies, the methylation and deamination resulting from the CG to TG transition could generate the CATG motifs attractive to p53 for binding *in vivo* [126]. Some *Alu* elements contain negative calcium response elements [127, 128], cholesteryl esterase transferase response elements [129–131], and so forth. In addition, *Alu* elements with conserved noncoding elements in mammalian genomes were found to be the enhancers, such as that of *FGF8* and *SATB2* genes, and are involved in brain development [132]. An *Alu* element in the last intron of T-cell-specific *CD8α* gene was found to serve as an enhancer with GATA3, bHLH protein, and LYF1 binding motifs [133].

Experimentally, some* Alu *elements were shown to host various kinds of nuclear hormone response elements (HREs) (Figure 1), including the retinoic acid response element (RARE) [134–137], the estrogen response element (ERE) [138–141], the thyroid hormone response element (TRE) [137], and the vitamin D receptor binding element (VDRE) [142]. An* Alu* element containing the vitamin D receptor binding element in the promoter of* CAMP *gene mediates the vitamin D-cathelicidin pathway and becomes a key component of a novel innate immune response of human to infection [142].* Alu*-DR (direct repeat) elements provide the binding sites for hepatocyte nuclear factor 4 alpha (HNF4*α*) which activates the transcription of a list of target genes [143]. Laperriere calculated the frequencies of all DR motifs and found that over 100,000 consensus DR elements are present in* Alu *repeats, in particular* AluS*. Importantly, consensus* Alu*-DR2 elements arose predominantly via deamination of a methylated CpG dinucleotide [134]. By acquiring specific point mutations, some* Alus* gain the palindromic inserted repeats separated by three base pairs and acquired the ability to function as estrogen receptor-dependent enhancers [144, 145].

As shown in Figure 1, the distribution of various binding motifs shares the same sequences in* Alu *repeats, and, additionally, most RNA Pol IITF binding motifs cluster in the RNA Pol III promoter sequences, which provoke the thinking of the competition and cooperation among these binding proteins. On the other hand, how RNA Pol II and Pol III transcription machineries utilize* Alu *repeats in different tissues (such as SABP in sperm versus p68 in somatic cells) and under different microenvironments to recruit different transcription factors remains a mystery. Shankar found that a progressive loss of the Pol III transcriptional potential was observed with concomitant accumulation of Pol II regulatory sites [146], while another study also found that Pol II is present at the majority of genomic loci which are bound by Pol III [122], and Pol III occupancy is remarkably correlated to the levels of nearby PolII, active chromatin, and CpG content [121]. All of the above indicate that the regulatory function of* Alu *sequences is involved in the balanced activity between Pol II and Pol III. The methylation statuses of* Alu *elements would certainly determine the accessibility of* Alu *containing* cis-*elements and have a great impact on the binding of transcriptional factors. However, no genome-wide data has been generated so far to explore the* Alu *methylation patterns and the binding profiles of various factors on* Alu *elements.

## 6. Concluding Remarks

Decades of studies have demonstrated that* Alu* elements play important roles in the genome, from their contribution to the epigenome to their occurrence in a significant fraction of the human transcriptome. The time when they were considered to be nothing but junk is certainly long gone. DNA methylation is one of the key mechanisms controlling* Alu *expression. The dynamic* Alu *methylation during development and aging suggests* Alu *elements and their transcripts may be involved in the cell differentiation and tissue development. The methylation levels of a subset of* Alu *elements have been associated with tumor malignancy and aggressiveness. This indicates a potential biological and clinical relevance of the methylation of some* Alu *elements during tumorigenesis and progression. Since the interactions between genes and* Alus* are multidimensional, the impact of such epigenetic alterations on* Alu *elements may be multifold. Future integrative* Alu *epigenomic and transcriptomic studies will further elucidate the functional aspects of* Alu *elements and their transcripts.
